# A Note on the Solutions of Some Nonlinear Equations Arising in Third-Grade Fluid Flows: An Exact Approach

**DOI:** 10.1155/2014/109128

**Published:** 2014-03-17

**Authors:** Taha Aziz, F. M. Mahomed

**Affiliations:** ^1^Differential Equations, Continuum Mechanics and Applications, School of Computational and Applied Mathematics, University of the Witwatersrand, Wits 2050, South Africa; ^2^School of Mathematics and Statistics, University of New South Wales, Sydney, NSW 2052, Australia

## Abstract

In this communication, we utilize some basic symmetry reductions to transform the governing nonlinear partial differential equations arising in the study of third-grade fluid flows into ordinary differential equations. We obtain some simple closed-form steady-state solutions of these reduced equations. Our solutions are valid for the whole domain [0,∞) and also satisfy the physical boundary conditions. We also present the numerical solutions for some of the underlying equations. The graphs corresponding to the essential physical parameters of the flow are presented and discussed.

## 1. Introduction

The study of non-Newtonian fluids involves the modelling of materials with dense molecular structure such as polymer solutions, slurries, blood, and paints. These material exhibit both viscous behavior like liquids and elastic behavior like solids. Therefore, the understanding of the complex behavior and properties of non-Newtonian fluids is crucial these days. The problems dealing with the flow of non-Newtonian fluids have several technical applications in engineering and industry. Some of them are the extraction of crude oil from petroleum products, oil and gas well drilling, food stuff, extrusion of molten plastic, paper and textile industry, and so on. The flow properties of non-Newtonian fluids are quite different from those of the Newtonian fluids. Therefore, in practical applications, one cannot replace the behavior of non-Newtonian fluids with Newtonian fluids and it is very important to analyze and understand the flow behavior of non-Newtonian fluids in order to obtain a thorough understanding and improve utilization in various manufactures.

In the past couple of years, many nonlinear problems dealing with non-Newtonian fluids have been taken into account. Some important studies dealing with the flow of non-Newtonian fluids are due to the studies of Abd-el-Malek et al. [1], Ariel et al. [2], Chen et al. [3], C. Fetecau and C. Fetecau [4], Hayat et al. [5, 6], Rajagopal et al. [7], Fosdick and Rajagopal [8], Rajagopal [9], and many studies thereafter. Some researchers have utilized the numerical approaches [10–12] to tackle these sorts of problems and several nonlinear problems have recently been solved by the homotopy analysis method (HAM) [13–15]. The purpose of this study is to discuss some nonlinear equations arising in the study of third grade fluids [16–18] analytically in a simplest possible way instead of using the HAM or other difficult techniques. The third-grade fluid model represents a further although inconclusive attempt to study the physical structure of non-Newtonian fluids. This model is known to capture the very interesting phenomena like die-swell, rod climbing effect [19], shear thinning, and the shear thickening that many other non-Newtonian models do not exhibit. We consider three different problems, namely, (i) flow of a third grade fluid over a flat rigid plate within porous medium, (ii) flow of a third-grade fluid in a porous half space with suction/injection effects, and (iii) magnetohydrodynamic (MHD) flow of a third-grade fluid in a porous half space. We solve all of these problems by imposing the relevant boundary conditions to make the problems well posed.

The main focus in this work is to construct a class of closed-form solutions for boundary value problems for nonlinear diffusion equations arising in the study of non-Newtonian third- grade fluids thorough a porous medium with the principle of Lie group method for differential equations. We also obtain the numerical solutions for the underlying models to show how various physical parameters play their part in determining the properties of the flow.

## 2. Geometry of the Problems

We consider a Cartesian coordinate system *OXYZ* with the *y*-axis in the vertically upward direction. The third-grade fluid occupies the porous space *y* > 0 and is in contact with an infinite moved plate at *y* = 0. Since the plate is infinite in the *XZ*-plane and therefore all the physical quantities except the pressure depend on *y* only. The flow is caused by the motion of the plate in its own plane with an arbitrary velocity. Far away from the plate, the fluid will be considered to be at rest. We have taken three different problems on the same flat plate geometry.

## 3. Problems to Be Investigated

### 3.1. Unsteady Flow of Third-Grade Fluid over a Flat Rigid Plate with Porous Medium

By following the methodology of References. [20, 21], the equation of motion for the unsteady flow of third-grade fluid over the rigid plate with porous medium is

(1) ρ∂U∂t=μ∂2U∂y2+α1∂3U∂y2∂t+6β3(∂U∂y)2∂2U∂y2−ϕκ[μ+α1∂∂t+2β3(∂U∂y)2]U.

Here *U* is the velocity component, *ρ* is the density, *μ* the coefficient of viscosity, *α*_1_ and *β*_3_ are the material constants (for details on these material constants and the conditions that are satisfied by these constants, the reader is referred to [8]), *ϕ* the porosity and *κ* the permeability of the porous medium.

In order to solve (1) mentioned above, boundary conditions are specified as follows:

(2) $$\begin{array}{c} U\left(0,t\right)=U_{0}V\left(t\right),\;t>0, \end{array}$$

(3) $$\begin{array}{c} U\left(\infty ,t\right)=0,\;t>0, \end{array}$$

(4) $$\begin{array}{c} U\left(y,0\right)=0,\;y>0, \end{array}$$

where *U*_0_ is the reference velocity. The first boundary condition (2) is the no-slip condition and the second boundary condition (3) says that the main stream velocity is zero. This is not a restrictive assumption since we can always measure velocity relative to the main stream. The initial condition (4) indicates that the fluid is initially at rest.

On introducing the nondimensional quantities

(5) $$\begin{array}{c} \overset{-}{U}=\frac{U}{U_{0}},\;\;\overset{-}{y}=\frac{U_{0}y}{\nu },\;\;\overset{-}{t}=\frac{U_{0}^{2}t}{\nu },\\ \overset{-}{\alpha }=\frac{\alpha_{1}U_{0}^{2}}{\rho \nu^{2}},\;\;\overset{-}{\beta }=\frac{2\beta_{3}U_{0}^{4}}{\rho \nu^{3}},\;\;\frac{1}{\overset{-}{K}}=\frac{\phi \nu^{2}}{\kappa U_{0}^{2}}. \end{array}$$

Equation (1) and the corresponding initial and the boundary conditions take the form

(6) ∂U∂t=∂2U∂y2+α∂3U∂y2∂t+3β(∂U∂y)2∂2U∂y2−1K[U+α∂U∂t+βU(∂U∂y)2],

(7) $$\begin{array}{c} U\left(0,t\right)=V\left(t\right),\;t>0, \end{array}$$

(8) $$\begin{array}{c} U\left(y,t\right)⟶0\;\text{as}\;\;y⟶\infty ,\;t>0, \end{array}$$

(9) $$\begin{array}{c} U\left(y,0\right)=0,\;y>0. \end{array}$$

For simplicity, we ignore the bars of the nondimensional quantities. We rewrite (6) as

(10) ∂U∂t=μ∗∂2U∂y2+α∗∂3U∂y2∂t+3γ(∂U∂y)2∂2U∂y2−γ∗U(∂U∂y)2−1K∗U,

where

(11) $$\begin{array}{c} \mu_{*}=\frac{1}{\left(1+\alpha /K\right)},\;\;\alpha_{*}=\frac{\alpha }{\left(1+\alpha /K\right)},\;\;\gamma =\frac{\beta }{\left(1+\alpha /K\right)},\\ \gamma_{*}=\frac{\beta /K}{\left(1+\alpha /K\right)},\;\;\frac{1}{K_{*}}=\frac{1/K}{\left(1+\alpha /K\right)}. \end{array}$$

We know that from the principal of Lie group theory that if a differential equation is explicitly independent of any dependent or independent variable, then this particular differential equation remains invariant under the translation symmetry corresponding to that particular variable. We notice that (10) is independent of *t*, so it is invariant under the Lie point symmetry generator **X** = ∂/∂*t*. So, by the theory of invariants, the solution of (10) corresponding to operator **X** is obtained by

(12) $$\begin{array}{c} \frac{dt}{1}=\frac{dy}{0}=\frac{dU}{0}, \end{array}$$

implyingthe steady-state solution given by

(13) $$\begin{array}{c} U=F\left(\eta \right),\;\text{where}\;\;\eta =y. \end{array}$$

Inserting (13) into (10) yields

(14) $$\begin{array}{c} \mu_{*}\frac{\partial^{2}F}{\partial \eta^{2}}+3\gamma {\left(\frac{\partial F}{\partial \eta }\right)}^{2}\frac{\partial^{2}F}{\partial \eta^{2}}-\gamma_{*}F{\left(\frac{\partial F}{\partial \eta }\right)}^{2}-\frac{1}{K_{*}}F=0, \end{array}$$

and the transformed boundary conditions are given by

(15) $$\begin{array}{c} F\left(0\right)=l_{1},\;\text{where}\;\;l_{1}\;\;\text{is a constant}\\ F\left(\eta \right)⟶0,\;\text{as}\;\;y⟶\infty . \end{array}$$

We have now transformed the governing nonlinear PDE (10) into a nonlinear ODE (14) as well as the boundary conditions (7)–(9) to the boundary conditions (15).

In order to solve (14) for *F*(*η*), we assume a solution of the form

(16) $$\begin{array}{c} F\left(\eta \right)=A\;\exp\left(B\eta \right), \end{array}$$

where *A* and *B* are the constants to be determined. Substituting (16) into (14), we obtain

(17) $$\begin{array}{c} \left(\mu_{*}B^{2}-\frac{1}{K_{*}}\right)+e^{2B\eta }\left(3\gamma A^{2}B^{4}-\gamma_{*}A^{2}B^{2}\right)=0. \end{array}$$

Separating (17) in the powers of *e* ^0^ and *e* ^2*Bη*^, we deduce

(18) $$\begin{array}{c} e^{0}:\;\mu_{*}B^{2}-\frac{1}{K_{*}}=0, \end{array}$$

(19) $$\begin{array}{c} e^{2B\eta }:\;3\gamma A^{2}B^{4}-\gamma_{*}A^{2}B^{2}=0. \end{array}$$

From (19), we find

(20) $$\begin{array}{c} B=\pm \frac{\sqrt{\gamma_{*}}}{\sqrt{3\gamma }}. \end{array}$$

We choose

(21) $$\begin{array}{c} B=-\frac{\sqrt{\gamma_{*}}}{\sqrt{3\gamma }}, \end{array}$$

so that our solution would satisfy the second boundary condition at infinity. Using the value of *B* in (18), we obtain

(22) $$\begin{array}{c} \frac{\gamma_{*}}{3\gamma }\mu_{*}-\frac{1}{K_{*}}=0. \end{array}$$

Thus, the solution for *F*(*η*) can be written as

(23) $$\begin{array}{c} U=F\left(\eta \right)=l_{1}\exp\left(-\frac{\sqrt{\gamma_{*}}}{\sqrt{3\gamma }}\eta \right), \end{array}$$

provided that condition (22) holds, where *A* = *l*_1_ by using the first boundary condition.

### 3.2. Unsteady Flow of Third-Grade Fluid over a Flat Porous Plate with Porous Medium

By employing the same geometry as we have explained in Section 2, we extend the previous problem by incorporating the effects of plate suction or injection. We provide the closed-form solution of the problem by reducing the governing nonlinear PDE into an ODE with the aid of Lie groups.

The present analysis deals with a porous plate with suction or injection and thus the velocity field is given by

(24) $$\begin{array}{c} V=\left[U\left(y,t\right),-W_{0},0\right], \end{array}$$

where *W*_0_ > 0 denotes the suction velocity and *W*_0_ < 0 indicates blowing velocity. One can see that the incompressibility constraint is identically satisfied; that is,

(25) $$\begin{array}{c} \mathrm{div}V=0. \end{array}$$

So, the unsteady flow through a porous medium and over a porous plate in the absence of the modified pressure gradient takes the form

(26) ρ[∂U∂t−W0∂U∂y] =μ∂2U∂y2+α1∂3U∂y2∂t  −α1W0∂3U∂y3+6β3(∂U∂y)2∂2U∂y2−ϕκ  ×[μ+α1∂∂t−α1W0∂∂y+2β3(∂U∂y)2]U.

The boundary conditions remain the same as given in (2)–(4). Defining the nondimensional parameters as

(27) $$\begin{array}{c} \overset{-}{U}=\frac{U}{U_{0}},\;\;\overset{-}{y}=\frac{U_{0}y}{\nu },\\ \overset{-}{t}=\frac{U_{0}^{2}t}{\nu },\;\;\overset{-}{\alpha }=\frac{\alpha_{1}U_{0}^{2}}{\rho \nu^{2}},\\ \overset{-}{\beta }=\frac{2\beta_{3}U_{0}^{4}}{\rho \nu^{3}},\;\;\frac{1}{\overset{-}{K}}=\frac{\phi \nu^{2}}{\kappa U_{0}^{2}},\;\;\overset{-}{W}=\frac{W_{0}}{U_{0}}. \end{array}$$

Equation (28) becomes

(28) [∂U∂t−W∂U∂y]=∂2U∂y2+α∂3U∂y2∂t−αW∂3U∂y3+3β(∂U∂y)2∂2U∂y2−1K×[U+α∂U∂t−αW∂U∂y+βU(∂U∂y)2],

with the boundary conditions taking the form as given in (7)–(9). We rewrite (28) as

(29) ∂U∂t=μ∗∂2U∂y2+α∗∂3U∂y2∂t−α∗W∂3U∂y3+3γ(∂U∂y)2∂2U∂y2−γ∗U(∂U∂y)2+W∂U∂y−1K∗U,

where *μ*_∗_, *α*_∗_, *γ*_∗_, *γ*, and 1/*K*_∗_ are defined in (11). Now, we have to solve (29) subject to the boundary conditions (7)–(9).

As it can be seen, (29) is invariant under the time-translation symmetry generator **X** = ∂/∂*t*. The invariant solution corresponding to ∂/∂*t* is the steady-state solution given by

(30) $$\begin{array}{c} U=G\left(\eta \right),\;\text{where}\;\;\eta =y. \end{array}$$

Using (30) into (29) yields

(31) μ∗d2Gdη2−α∗Wd3Gdη3+3γ(dGdη)2d2Gdη2  −γ∗G(dGdη)2+WdGdη−1K∗G=0,

with the transformed boundary conditions

(32) $$\begin{array}{c} G\left(0\right)=l_{2},\;\text{where}\;\;l_{2}\;\;\text{is a constant,}\\ G\left(\eta \right)⟶0,\;\;\frac{dG}{d\eta }⟶0\;\text{as}\;\;y⟶\infty . \end{array}$$

Following the same methodology as adopted in solving the previous problem, (31) admits an exact solution of the form (which we require to be zero at infinity due to the condition *G*(*∞*) = 0)

(33) $$\begin{array}{c} U=G\left(\eta \right)=l_{2}\exp\left(-\frac{\sqrt{\gamma_{*}}}{\sqrt{3\gamma }}\eta \right), \end{array}$$

provided that

(34) $$\begin{array}{c} \frac{\gamma_{*}}{3\gamma }\mu_{*}+\alpha W\sqrt{\frac{\gamma_{*}}{3\gamma }}\frac{\gamma_{*}}{3\gamma }-\sqrt{\frac{\gamma_{*}}{3\gamma }}W-\frac{1}{K_{*}}=0. \end{array}$$

Note that, if we set *W* = 0, we can recover the condition given in (22).

### 3.3. Unsteady Magnetohydrodynamic (MHD) Flow of Third-Grade Fluid over a Flat Porous Plate with Porous Medium

In this problem, we extend the previous one by considering the fluid to be electrically conducting under the influence of a uniform magnetic field applied transversely to the flow.

The unsteady MHD flow of a third-grade fluid in a porous half space with plate suction/injection is governed by

(35) ρ[∂U∂t−W0∂U∂y]=μ∂2U∂y2+α1∂3U∂y2∂t−α1W0∂3U∂y3+6β3(∂U∂y)2∂2U∂y2−ϕκ×[μ+α1∂∂t−α1W0∂∂y+2β3(∂U∂y)2]×U−σB02U,

where *σ* is the electrical conductivity and **B**_0_ the uniform applied magnetic field. Again the boundary conditions remain the same as given in (2)–(4). We define the dimensionless parameters as

(36) $$\begin{array}{c} \overset{-}{U}=\frac{U}{U_{0}},\;\;\overset{-}{y}=\frac{U_{0}y}{\nu },\;\;\overset{-}{t}=\frac{U_{0}^{2}t}{\nu },\;\;\overset{-}{\alpha }=\frac{\alpha_{1}U_{0}^{2}}{\rho \nu^{2}},\\ \overset{-}{\beta }=\frac{2\beta_{3}U_{0}^{4}}{\rho \nu^{3}},\;\;\frac{1}{\overset{-}{K}}=\frac{\phi \nu^{2}}{\kappa U_{0}^{2}},\\ {\overset{-}{M}}^{2}=\frac{\sigma B_{0}^{2}\nu }{\rho U_{0}^{2}},\;\;\overset{-}{W}=\frac{W_{0}}{U_{0}}. \end{array}$$

Equation (26) takes the form

(37) [∂U∂t−W∂U∂y]=∂2U∂y2+α∂3U∂y2∂t−αW∂3U∂y3+3β(∂U∂y)2∂2U∂y2−1K[U+α∂U∂t−αW∂U∂y+βU(∂U∂y)2]−M2U.

We rewrite (37) as

(38) ∂U∂t=μ∗∂2U∂y2+α∗∂3U∂y2∂t−α∗W∂3U∂y3+3γ(∂U∂y)2∂2U∂y2−γ∗U(∂U∂y)2+W∂U∂y−(1K∗+M∗2)U,

where *μ*_∗_, *α*_∗_, *γ*_∗_, *γ*, and 1/*K*_∗_ are defined in (11) and

(39) $$\begin{array}{c} M_{*}^{2}=\frac{M^{2}}{\left(1+\alpha /K\right)}. \end{array}$$

Now, we need to solve (38) subject to the boundary conditions (7)–(9). Since (38) is invariant under the time-translation symmetry generator **X** = ∂/∂*t*, the invariant solution corresponding to ∂/∂*t* is the steady-state solution. Consider

(40) $$\begin{array}{c} U=H\left(\eta \right),\;\text{where}\;\;\eta =y. \end{array}$$

Invoking (40) in (38) yields

(41) μ∗d2Hdη2−α∗Wd3Hdη3+3γ(dHdη)2d2Hdη2  −γ∗H(dHdη)2+WdHdη−(1K∗+M∗2)H=0,

with the transformed boundary condition

(42) $$\begin{array}{c} H\left(0\right)=l_{3},\;\text{where}\;\;l_{3}\;\;\text{is a constant},\\ H\left(\eta \right)⟶0,\;\;\frac{dH}{d\eta }⟶0\;\text{as}\;\;y⟶\infty . \end{array}$$

Utilizing the same method adopted to solve the first problem, (41) admits an exact solution of the form (which we require to be zero at infinity due to the second boundary condition)

(43) $$\begin{array}{c} U=H\left(\eta \right)=l_{3}\exp\left(-\frac{\sqrt{\gamma_{*}}}{\sqrt{3\gamma }}\eta \right), \end{array}$$

provided that

(44) $$\begin{array}{c} \frac{\gamma_{*}}{3\gamma }\mu_{*}+\alpha W\sqrt{\frac{\gamma_{*}}{3\gamma }}\frac{\gamma_{*}}{3\gamma }-\sqrt{\frac{\gamma_{*}}{3\gamma }}W-\frac{1}{K_{*}}-M_{*}^{2}=0. \end{array}$$

Note that, if we set *W* = *M*_∗_ = 0, we can recover the previous two conditions given in (22) and (34).

**Remark 1**

We note that the similarity solutions (23), (33), and (43) are the same, but the imposing conditions (22), (34), and (44) under which these solutions are valid are different. These solutions do show the effects of the third-grade fluid parameters *γ* and *γ*_∗_ on the flow. However, they do not directly contain the term which is responsible for showing the effects of suction/blowing and magnetic field on the flow. The imposing conditions do contain the magnetic field and suction/blowing parameters. Thus, these closed-form solutions are valid for the particular values of the suction/blowing and the magnetic field parameters. To show the effects of these interesting phenomena, we have solved the reduced (14), (31), and (41) with the boundary conditions (15), (32), and (42) numerically using the powerful numerical solver NDSolve in Mathematica. These numerical solutions are plotted in Figures 2–4 against the emerging parameters of the flow.

## 4. Analysis and Discussion

**Figure 1:**
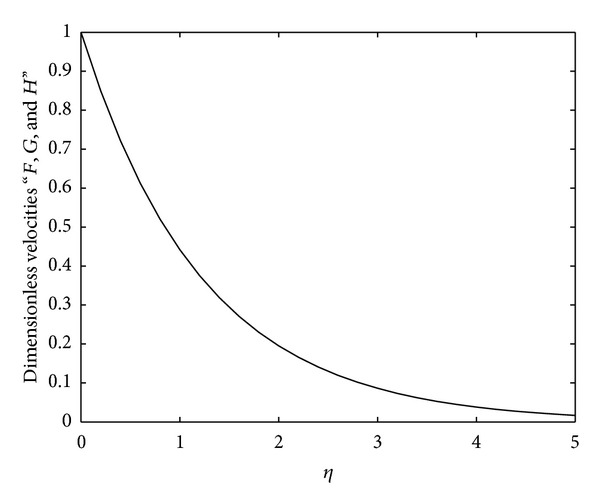
Profile of the analytical solutions *F*, *G*, and *H* as a function of dimensionless coordinate *η*, where we have chosen *γ* = 1.5, *γ*_∗_ = 1, and *l*_1_ = *l*_2_ = *l*_3_ = 1.

**Figure 2:**
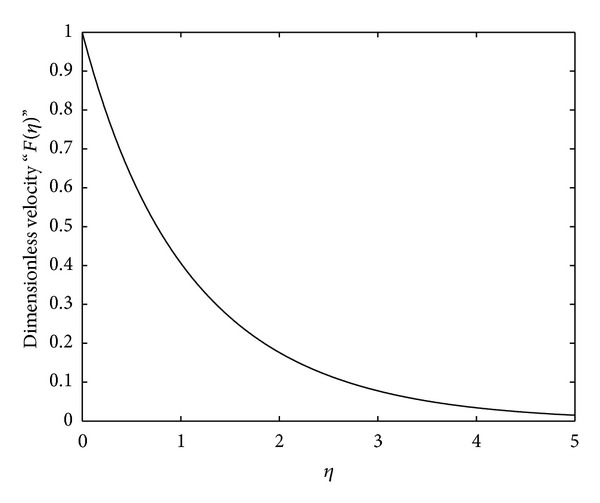
Numerical solution of ODE (14) subject to the boundary conditions (15), where we have chosen *γ* = 0.5, *γ*_∗_ = 0.75, *μ*_∗_ = 1.5, and *K*_∗_ = *α*_∗_ = 1.

**Figure 3:**
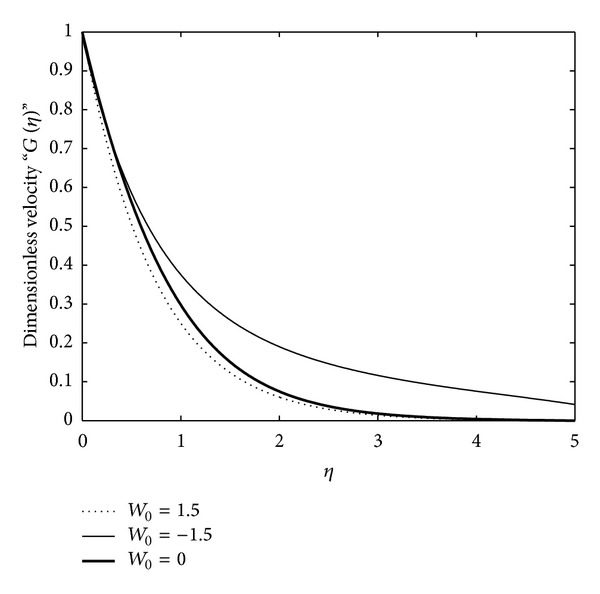
Numerical solution of ODE (31) subject to the boundary conditions (32) for varying values of *W*_0_, where we have chosen *γ* = 1.5, *γ*_∗_ = 1, *μ*_∗_ = 0.5, and *K*_∗_ = *α*_∗_ = 1.

**Figure 4:**
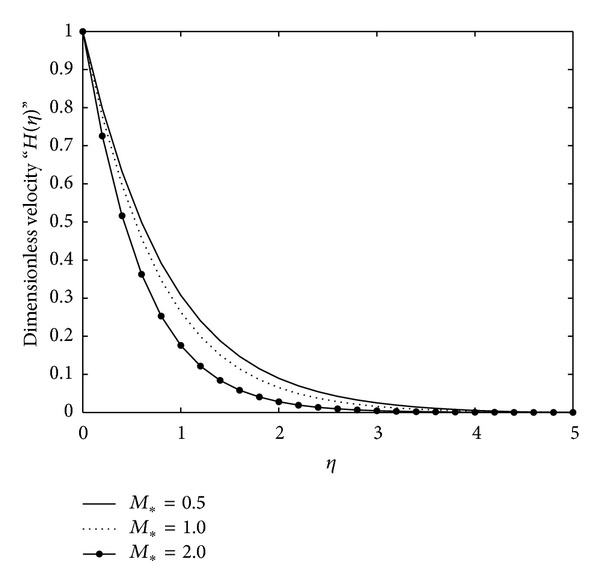
Numerical solution of ODE (41) subject to the boundary conditions (42) for varying values of *M*_∗_, where we have chosen *γ* = 1.5, *γ*_∗_ = 0.5, *μ*_∗_ = 0.5, *W*_0_ = 0.75, and *K*_∗_ = *α*_∗_ = 1.

**Figure 5:**
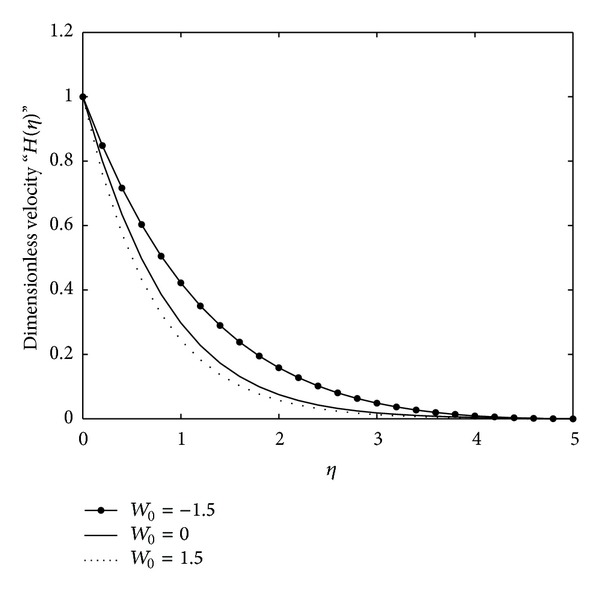
Numerical solution of ODE (41) subject to the boundary conditions (42) for varying values of *W*_0_, where we have chosen *γ* = 1.5, *γ*_∗_ = 0.5, *μ*_∗_ = 0.5, *M*_∗_ = 0.5, *K*_∗_ = 0.75, and *α*_∗_ = 1.

In order to analyze the behavior and properties of some of the essential physical parameters of the flow, we have plotted Figures 1–5.

In Figure 1, the closed-form solutions (23), (33), and (43) are plotted. Since these solutions physically behave in the same way, the restrictive conditions (22), (34), and (44) on the parameters under which these solutions are valid differ from each other. Therefore, the behavior of these solutions is indistinguishable in the graph.

In Figure 2, the numerical solution of the reduced ODE (14) is given. This numerical behavior of the velocity is exactly the same as observed previously in Figure 1 for the analytical solutions that velocity is a decreasing function of the dimensionless parameter *η*.

To examine the influence of plate suction/injection on the flow, Figures 3 and 5 have been plotted. In Figure 3, the reduced ODE (31) is plotted numerically for varying values of the parameter *W*_0_ and in Figure 5 the reduced ODE (41) is plotted numerically for the varying values of the parameter *W*_0_. The effect of the parameter *W*_0_ on the velocity field is exactly the same in both of these figures. From these figures, it is clearly indicated that for the case of suction (*W*_0_ > 0) the velocity field decreases as the boundary layer thickness and the effects of injection (*W*_0_ < 0) are totally opposite to those of suction. This is in agreement with what is expected physically.

Finally, the influence of the magnetic field on the structure on the velocity is analyzed in Figure 4. From the figure, it is observed quite clearly that with the increase of the Hartman number (magnetic field strength) *M*_∗_, the velocity field decreases. This is what we expect physically in this case as well.

## 5. Final Remarks

In this note, we have presented closed-form solutions for some nonlinear problems which describe the phenomena of third-grade fluids. In each case, the governing nonlinear PDEs reduced to nonlinear ODEs by using the Lie point symmetry (which is translation) in the *t* direction. The reduced ODEs are then solved analytically by employing a very simple approach and also have been solved numerically to show the effects of some of the interesting emerging parameters of the flow. The method of solution that we have used here is helpful for solving a wide range of nonlinear problems in a simple way instead of using other difficult techniques.
